# Antimicrobial Activity of Diffusible and Volatile Metabolites Emitted by *Beauveria bassiana*: Chemical Profile of Volatile Organic Compounds (VOCs) Using SPME-GC/MS Analysis

**DOI:** 10.3390/plants12152854

**Published:** 2023-08-03

**Authors:** Ippolito Camele, Sadeek A. Sadeek, Rocco Racioppi, Hazem S. Elshafie

**Affiliations:** 1 School of Agricultural, Forestry, Food and Environment al Sciences, University of Basilicata, Viale dell’Ateneo Lucano 10, 85100 Potenza, Italy; 2 Department of Chemistry, Faculty of Science, University of Zagazig, Zagazig 44519, Egypt; s_sadeek@zu.edu.eg; 3 Department of Sciences, University of Basilicata, Viale dell’Ateneo Lucano 10, 85100 Potenza, Italy; rocco.racioppi@unibas.it

**Keywords:** biocontrol, natural products, phytopathogens, microbial metabolites, entomopathogens, endophytic fungi

## Abstract

The genus *Beauveria* includes important entomopathogenic and endophytic fungi; among them, *Beauveria bassiana* is the most studied species. However, there is little knowledge regarding their antimicrobial activity. The current research has been conducted to evaluate the in vitro antagonistic activity of *B. bassiana* and the antimicrobial efficacy of its *Exo* and *Endo* metabolites against *Bacillus cereus, B. megaterium, Clavibacter michiganensis* (Gram positive bacteria, G+ve), *Xanthomonas campestris, Pseudomonas aeruginosa* and *P. fluorescence* (Gram negative bacteria, G−ve). In addition, solid-phase microextraction (SPME) was coupled with Gas Chromatography-Mass Spectrometry (GC/MS) to qualitatively measure the volatile organic compounds’ (VOCs) metabolic profile of the most efficient studied isolate of *B. bassiana*. The obtained results showed that the isolate UniB2439-3 has a promising antibacterial effect against most of the studied target bacteria. An SPME-GC/MS analysis of VOCs revealed the presence of ethanol, butanal,2-methyl, 2,4-dimethyl-1-heptene, octane, 4-methyl and β-elemene as the dominant bioactive compounds. The results demonstrated that the efficient isolate of *B. bassiana* can be potentially used as a biocontrol agent against several bacteria, especially G+ve ones.

## 1. Introduction

Genus *Beauveria* includes entomopathogenic and endophytic fungi, which are widespread in different habitats [1,2,3]. Furthermore, many researchers have reported that fungi in the genus *Beauveria* can produce enzymes for biotransformation and biodestructors [4,5,6]. Some species of this genus, such as *B. bassiana* and *B. brongniartii,* are able to produce mycoinsecticides [7]. *B. bassiana* is also a beneficial microorganism (BM) and endophytic fungus (EF) in several crops and is commonly known as biological control agent against a variety of agricultural pests [3,8,9,10]. The application of *B. bassiana* has many advantages, such as being a form of eco-friendly management compared to chemical pesticides, and being harmless to human health [8,10,11,12]. For decades, several scientists have reported the importance of *B. bassiana* in reducing a range of nuisance insects, where it can induce direct insect mortality [2,13,14] and reach a 90% reduction in life-time fecundity [15].

A recent study, conducted by Barra-Bucarei et al. [16] to evaluate the colonization ability of native endophytes of different strains of *B. bassiana* and their antifungal effect against *Botrytis cinerea* in tomato and chili pepper, concluded that all studied strains had significant in vitro antagonism against *B. cinerea.* The same study reported that the native strains of *B. bassiana* were able to colonize tomato and chili pepper tissues and provided important levels of antagonism against *B. cinerea* [16].

Sinno et al. [10] reported that different isolates of *B. bassiana* have plant-growth-promoting effects (PGP) and are a protective agent for tomato plants against *B. cinerea*, *Alternaria alternata,* the pest aphis, and *Macrosiphum euphorbiae* [10].

The results showed that some studied isolates were able to control the two phytopathogens, and one isolate was also able to promote plant growth [10]. The antibacterial activity of a crude ethyl acetate extract of *B. bassiana* against some aerobic pathogenic bacteria was tested by Parine et al. [17]. The results explained that the extract of *B. bassiana* possesses a strong inhibitory activity against many of the tested species, especially *Bacillus megaterium*, *B. subtilis*, *B. sphaericus* and *Escherichia coli* [17]. It showed a moderate effect against *Micrococcus luteus*, *Pseudomonas aeruginosa* and a low effect against *Streptococcus pyogenes* and *Chromobacterium violaceum* [17]. In another study, the application of conidia of *B. bassiana* protected tomato and potato seedlings from the damping-off disease caused by the soil-borne pathogen *Rhizoctonia solani* [18,19]. 

Recently, there is a large amount of interest in discovering natural-substances-based plants or those with microbe origins that have an antimicrobial effect [20,21]. However, the newly discovered natural substances should be evaluated for safety to avoid any possible negative health impact [22,23]. In addition, the discovery of possible natural alternatives to the excessive use of synthetic chemicals, decreasing the environmental hazards and avoiding the appearance of new microbial strains that are resistant to common microbicide compounds, should be highly considered [18,24]. 

There is little information regarding the bioactive metabolites with antimicrobial activity produced by either diffusable or volatile *B. bassiana* or their mechanism(s) of action regarding either their antimicrobial or plant-growth-promotion effects. A recent study conducted by Wang et al. [25] reported that *B. bassiana* produces a variety of toxins, such as beauvericin, bassianin, bassianolide, beauverolides, tenellin, oosporein, and oxalic acid, which enable *B. bassiana* to invade, parasitize, and destroy host tissues. Therefore, the precise chemical characterization and determination of the main bioactive single substances of *B. bassiana* will certainly aid in understanding its biological importance. In addition, the details of the chemical constituents of *B. bassiana* will undoubtedly have various applications, such as controlling plant diseases, taking into consideration the heavy reliance on chemicals that are extremely harmful to the environment as well as plants, animals, and human health. Furthermore, the insecticidal effects of *B. bassiana* have also been extensively studied, while their antifungal or antibacterial effects have received less attention.

Different volatiles are produced in huge quantities by a number of microorganisms. The volatile molecules, which can be both organic (VOCs) and inorganic, are crucial for such an environment, since they have the power to affect both beneficial and harmful microbes [26]. The significance of studying microbial volatile compounds is due to the fact that one of the typical strategies of inter- and intra-organismal communication is due to their production of volatile substances [26].

The main objective of this research is to study the chemical composition of the principal volatile organic compounds (VOCs) of *B. bassiana*. Hence, the chemical composition of *B. bassiana* metabolites will aid in the detection and differentiation of this species from others. The full identification of its metabolite profile can aid in its utilization in industrial, agricultural, and pharmaceutical fields. In addition, in this research, we will expand the possible benefits of *B. bassiana* against new non-reported target phytopathogens. The aims of the current research were to: (i) evaluate the antagonistic activity of five isolates of *B. bassiana* against some bacteria; (ii) investigate the in vitro antimicrobial activity of diffusible and volatile metabolites produced by the most efficient isolate; and (iii) chemically characterize VOCs produced by the most efficient isolate using SPME-GC/MS analysis.

## 2. Results

### 2.1. Molecular Identification of the Studied Isolates of Beauveria

The PCR amplification for β-tubulin genes with Bt2a/Bt2b produced, for each gDNA extracted from the above five isolates (UniB2439-1, UniB2439-2, UniB2439-3, UniB2439-4, and UniB2439-5), amplicons with a nucleotide length of about 330 bp. The DNA extracted from the same five isolates and amplified with ITS5/ITS4 for rRNA produced amplicons with a nucleotide length of about 600 bp. No amplification was observed in the case of the negative control. The amplicons were directly sequenced (BMR Genomics, Padova, Italy), and the obtained sequences were compared with those available in the GenBank nucleotide archive, showing a high similarity percentage (97.29%) with the sequences AB829899, AB829898, and CP045886.1, and those available for *B. bassiana* in the NCBI database using Basic Local Alignment Search Tool software (BLAST) (Bethesda, Rockville Pike, MD, USA) [27]. The five obtained sequences were deposited in the NCBI GeneBank with accession numbers FR989662–FR989666. The phylogenetic analysis confirmed the identification of the five studied isolates as *B. bassiana* (Figures S1 and S2).

### 2.2. Antagonistic Activity of B. bassiana Isolates 

**Figure 1:**
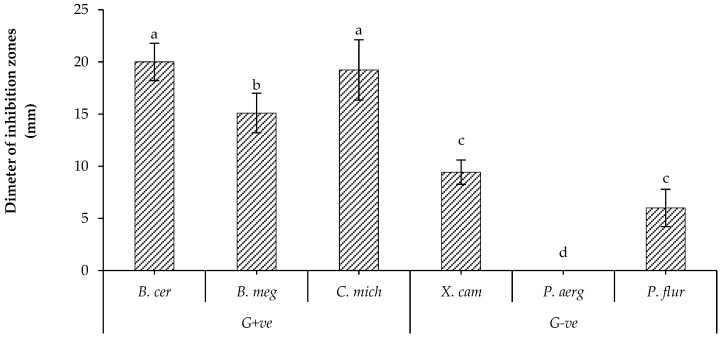
Antagonistic activity of *B. bassiana* UniB2439-3. Bars with different letters are significantly different at *p* < 0.05. Data for each bar are expressed as the mean of three replicates ± SDs.

The preliminary results showed that all tested isolates of *B. bassiana* had antagonistic effects against most tested bacterial strains, as illustrated in Table S1. UniB2439-3 was the most efficient isolate. In fact, this isolate showed the most significant effect against *Bacillus cereus* and *Clavibacter michiganensis*, a moderate effect against *B. megaterium* and a low effect against *Xanthomonas campestris* and *Pseudomonas fluorescens*. This isolate did not show any activity against *P. aeruginosa* (Figure 1). Therefore, the UniB2439-3 isolate was selected for further biological and chromatographic analyses.

### 2.3. Antimicrobial Activity of Exo- and Endo-Diffusible Metabolites 

The antimicrobial activity of extracted metabolites was determined following the disc-diffusion method. The obtained results for the metabolites extracted from the selected isolate of *B. bassiana* UniB2439-3 showed that extracellular metabolites (*Exo*-ME) were more able to inhibit the growth of most tested bacterial strains than the endocellular (*Endo*-ME) (Table 1). In particular, *Exo*-ME showed the most significant activity against *C. michiganensis*. In addition, both extracts showed equal activity against *X. campestris*, whereas only *Endo*-ME showed antibacterial activity against *P. aeruginosa*. On the other hand, *Endo*-ME was not active against *B. cereus* or *C. michiganensis*. 

### 2.4. Antibacterial Activity of Volatiles Metabolites

The in vitro antibacterial activity of the volatile metabolites eventually emitted by *B. bassiana* was evaluated against both the grown-visible colonies (GVC) and aqueous suspension (AQS) of each tested bacterial strain. The results of an in vitro bioactivity assay demonstrated that the studied isolate of *B. bassiana* (UniB2439-3) produced bioactive volatile metabolites that were able to significantly reduce the growth of tested bacterial strains compared to tetracycline (positive control). In particular, the efficacy of the produced volatile substances was high against the AQS of all tested bacterial strains, higher than GVC (Table 2). In addition, the highest antibacterial activity was observed in the case of GVC against *B. megaterium* (G+ve) and *P. fluorescens* (G−ve), estimated at 77.5 and 52.5%, respectively. On the other hand, the highest antibacterial activity in the case of AQS was observed against *B. megaterium* (G+ve) and *P. aeruginosa* (G−ve), estimated at 92.0 and 87.5%, respectively.

### 2.5. SPME-GC/MS Analysis of VOCs

**Figure 2:**
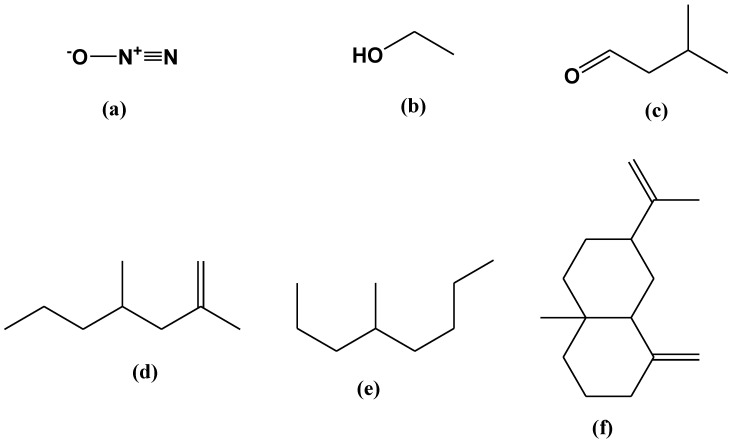
Chemical structures of the main VOCs identified by the SPME analysis: (**a**) nitrous oxide; (**b**) ethanol; (**c**) butanal,3-methyl; (**d**) 2,4-dimethyl-1-heptene; (**e**) octane, 4-methyl and (**f**) β-elemene.

A GC-MS analysis of the VOCs produced by *B. bassiana* UniB2439-3 was illustrated in Figure S3. In Table 3, all detected volatile compounds identified from *B. bassiana* UniB2439-3 are listed. The most dominant principal compounds, followed by their relative area percentage (R.A.%), are as follows: (i) nitrous oxide (27.57%), (ii) ethanol (4.69%), (iii) butanal, 3-methyl (1.32%), (iv) 2,4-dimethyl-1-heptene (0.63%), (v) octane, 4-methyl (1.99%), and (vi) β-elemene (6.98%) (Figure 2a–f). The mass spectra of the most abundant compounds are illustrated in Figures S4–S8. The resulting VOCs were not detected in the PDA culture used as a negative control (not inoculated with the fungus). 

The eventual fragmentation of the acquired volatile metabolites, as described here, is also shown in Table 3. Beauvericin was fragmented into diethyl phthalate with 90%, the major dominant constituent, carbon dioxide, and nitrous oxide. Bassianolide was converted to butanal, 3-methyl- with 81% or 1-butanol, 3-methyl- with 83%. Regarding bassianin, GC-MS analysis showed that this compound was converted into 2,4-Dimethyl-1-heptene with 90%. Beauveriolide was fragmented into butanal, 3-methyl with 81%, carbon dioxide and nitrous oxide. Regarding cyclosporine, the results demonstrated that this compound was fragmented into butanal, 2-methyl with 90%, butanal, 3-methyl with 81%, 1-butanol, 3-methyl with 83%, carbon dioxide and nitrous oxide. 

## 3. Discussion

Research has been conducted recently to overcome the multi-drug-resistant (MDR) microorganisms to different antibiotics and chemotherapeutic agents [29]. Hence, the search for new active and natural agents has attracted great interest, particularly for human health and environmental protection [30]. *Beauveria*, one of the most studied genera among entomopathogenic fungi, has various biological applications as a growth-promoting agent or insecticide [9,10,31,32]. 

The results revealed that both extracts are less effective than the control (tetracycline); nevertheless, they can be regarded as hopeful and prospective antimicrobial agents or as alternatives for synthetic pesticides. On the other hand, considering the higher activity of *Exo*-ME against *C. michiganensis* and the equal activity of both extracts against *X. campestris*, it would be beneficial to consider the potential synergistic effects of combining the two extracts in future studies.

On the other hand, Barra-Bucarei et al. [16] studied the antifungal activity of 10 native strains of *B. bassiana*, an endophyte for tomato and chili pepper, and observed that the majority of the studied native strains were able to colonize tomato and chili pepper tissues and showed a promising antagonistic effect against *B. cinerea.*

The capacity of *B. bassiana* to produce several volatile metabolites with possible antimicrobial effects is in agreement with previous bibliographic research investigating its antagonistic effect against several phytopathogens [9,17,33]. In fact, the bibliographic research revealed that the genus *Beauveria* produced some interesting metabolites, such as oosporein, beauvericin, bassianolide, bassianin, beauveriolide, bassiacridin and cyclosporine, with notable insecticide and antimicrobial actions [34,35,36,37,38,39,40]. 

Among the different bioactive metabolites produced by *B. bassiana*, several studies revealed that beauvericin and oosporein evidenced remarkable antibiotic and antifungal properties [39,40], which are probably involved in the microbial growth-inhibition observed in the bioassay presented in this study. Furthermore, Wang and Xu [41] reported that beauvericin was one of the active constituents of *B. bassiana* and confirmed to have antimicrobial activity and anti-tumor effects, especially against human leukemia. In another study, conducted by Manning and Wyatt [42], the results demonstrated that oosporein, extracted from the broth cultures of Beauveria and Chaetomium, has been identified as a toxic substance for plants and poultry.

Our findings from the SPME-GC/MS analysis showed that *B. bassiana* produces a variety of important VOCs, such as: (i) butanal, 3-methyl; (ii) 2,4-dimethyl-1-heptene; and (iii) octane, 4-methyl. These findings are in agreement with those of Chiron and Michelot [43], who explained that the main chemical groups released by fungi are alcohols (isomers of butanol, pentanol, and octanol), hydrocarbons, ketones, and terpenes [44].

The possible mechanism of volatile antimicrobial effects, in general, may be explained by the potential of volatiles to flow across a structure of soil gaps since they are active in both gaseous and liquid phases and have the potential of revolatization after flowing through water-saturated pores [26]. However, because of their high vapor pressure, volatiles mostly traverse through vapor diffusion. However, this process is regulated by the intrinsic chemical characteristics of each VOC and also the physicochemical characteristics of adjacent soil, which affect adsorption, desorption, and degradation.

In particular, 2,4-dimethyl-1-heptene showed antimicrobial activity, as reported by Mannaa and Kim [45]. In addition, 2,3,3-trimethyl-Octane, which is close to octane, 4-methyl, showed a higher docking energy than the commercial anti-inflammatory drug, as reported by Saravanakumar et al. [46]. Methyl-1-butanol was identified as one of the primary volatile chemicals released from active cultures of *Enterobacter agglomerans* [47]. Salih et al. [48] also reported that butanol, among the major constituents detected in *Coccoloba peltate*, showed notable antioxidant and cytotoxic effects.

Our obtained results also detected the presence of an important sesquiterpene compound identified as β-elemene (cyclohexane, 1-ethenyl-1-methyl-2,4-bis(1-methylethenyl), 1S-(1.alpha.,2.beta.,4. beta.) among the detected VOC substances from the studied *Beauveria* isolate. β-elemene was identified for the first time in 1994 in a dry rhizome extract from *Curcuma phaeocaulis*, *C. kwangsinensis*, and *C. wenyujing* [35]. In addition, β-elemene is also one of the common sesquiterpenes of several aromatic essential oils extracted from *Proteus vulgaris* [49]. β-elemene was also found in wild hops from Lithuania at levels up to 14% [50], and in notable amounts in the medical cannabis cultivar ‘bedropuur’ [51]. The same compound has notable antimicrobial activity against different pathogens, including *Mycobacterium tuberculosis,* as reported by Sieniawska et al. [52]. 

Generally, the mechanism of the antimicrobial activity of several terpenes is highly related to their lipophilic properties, which enable them to dissolve in the phospholipid layers of a microbial cell membrane [53]. Particularly, natural sesquiterpenes such as β-elemene, which originate from plants and microorganisms, showed promising antimicrobial activity [54,55]. A recent study conducted by Monga and Sharma [56] reported that β-elemene and R-limonene play an essential role in degrading the microbial cell wall, altering the expressions of *dprE1* and *clgR* genes, which are responsible for cell wall synthesis and cell membrane preservation, respectively. 

Some recent studies reported on the promising cytotoxic effects of β-elemene, which can inhibit cell proliferation, arrest cell cycle, and induce cell apoptosis or autophagy [57]. Β-elemene is one of the most promising inhibitors of the glycolysis rate-limiting enzyme, especially (PKM2), through its interference with tumor glycolysis, which is considered one of the most important recent strategies for treating tumors [58,59]. In fact, research has reported that inhibition of tumor growth and proliferation can be achieved by downregulating the expression of the PKM2 enzyme [60]. In addition, Pan et al. [61] pointed to the role of β-elemene in inhibiting breast cancer cell migration by converting dimer and tetramer forms of PKM2, inhibiting aerobic glycolysis, and reducing the utilization of glucose and the production of lactic acid for tumor cell growth.

## 4. Materials and Methods

### 4.1. Isolation, Culturing and Identification 

Five strains of *Beauveria bassiana* (UniB2439-1; UniB2439-2, UniB2439-3, UniB2439-4, UniB2439-5) were isolated from different rhizospheric soil samples of *Actinidia* spp. and identified based on their morphological features and molecular basis. For molecular identification, the total gDNA was extracted, and a partial part of β-tubulin and ribosomal DNA genes were amplified using the universal primers Bt2a (5′-GGTAACCAAATCGGTGCTGCTTTC-3′)/Bt2b (5′-ACCCTCAGTGTAGTGACCCTTGGC-3′) [62], and ITS5 (5′-GGAAGTAAAAGTCGTAACAAGG-3′)/ITS4 (5′-TCCTCCGCTTATTGATATGC-3′), which was used for amplifying the ribosomal DNA [63]. The obtained amplicons were sequenced and then analyzed using Basic Local Alignment Search Tool software (BLAST-USA). A partial phylogenetic analysis was carried out for the two amplified genes. The studied isolates were maintained as lyophils at 4 °C in the fungal collection of the School of Agricultural, Forestry, Food and Environmental Sciences (SAFE), University of Basilicata, Potenza, Italy. The subcultures were carried out on Sabouraud Dextrose Agar plus 1% yeast-extract (SDAY) nutrient media [5] and incubated at 22 ± 2 °C for 96 h [64]. 

### 4.2. Antagonistic Activity 

The antagonistic activity of the five studied isolates of *B. bassiana* was evaluated against some pathogenic bacteria. All tested isolates were obtained from the pure cultures conserved in the collection of SAFE and identified using morphological and molecular methods. The tested bacteria strains are listed in Table 4.

An antibacterial assay was carried out as described by Elshafie et al. [65]. A fungal disc of approximately 0.5 cm from the fresh PDA culture (96 h) of each studied isolate of *B. bassiana* was deposited in the center of the King B nutrient media (KB) Petri dish and incubated for 16 h at 22 ± 2 °C. Successively, a suspension of soft-agar (0.7%) of each tested bacteria at 10^8^ CFU/mL was sprayed over the plates using Eco-Spray Ecological Aerosol (Seidden Identification, Madrid, Spain). All plates were incubated at 30 °C for 24 h. Two KB plates inoculated only with each tested bacteria were used as a negative control. The experiment was run in triplicate, and the diameter of the inhibition zone was measured with a caliber and recorded as the mean  ±  SD (*n*  =  3). The antagonistic bacterial activity percentage (ABP%) was calculated using Equation (1):

ABP (%) = D.iz/D.ct × 100(1)

where ABP is the antagonistic bacterial activity; D.iz: is the diameter of inhibition zones in cm; D.ct: is the diameter of control plates in cm. 

### 4.3. Extraction of Secondary Metabolites

On the basis of the preliminary antagonistic assay, the most efficient isolate (UniB2439-3) of *B. bassiana* was selected for successive studies. For this purpose, 2 mL of the fungal suspension (10^6^ spores/mL) of the above isolate was used to inoculate 500 mL SDY broth nutrient media and then incubated for 7 days at 25 °C in agitation (180 rpm). Both *Exo*-ME and *Endo*-ME were extracted from the broth culture after the incubation period. 

For *Endo*-ME, the incubated broth culture was centrifuged at 40,000× *g* for 15 min, and the pellet (2 g) was collected, resuspended in 50 mL of Limonene (CAS 138-86-3- Aldrich, Steinheim, Germany) and shaken for 2 h; after this, the solvent was evaporated using the rotary-evaporator (Heidolph WB2000, Schwabach, Germany). The residue was resuspended in 2 mL of sterile distilled water (SDW), following the Solid Phase Extraction (SPE) method using a C-18 column (Thermo Scientific, Rockwood, TN, USA), and recovered using 1 mL methanol to reach the final original concentration of (20 mg/mL) [24]. 

For *Exo*-ME, the supernatant (250 mL) obtained from the above centrifugation step was filtered using 0.22 µm (syringe filter—hydrophilic, Minisart, Goettingen, Germany) and extracted using a separator funnel containing 250 mL ethyl acetate/ethanol (70:30; *v*/*v*) and shacked for 15 min. The organic phase was filtered through a filter paper (Whatman, Ø. 25 mm, Merck KGaA, Darmstadt, Germany) and evaporated using the rotary-evaporator. The dry residue (50 mg) was resuspended in 2 mL SDW, extracted through SPE using a C-18 column, and recovered using 1 mL methanol to reach the final original concentration of (16 mg/mL) [24].

### 4.4. Antibacterial Activity of Diffusible Metabolites 

The antibacterial activity of both metabolite extracts, compared with the same bacteria strains used for the initial antagonistic assay, is listed in Table 4.

Disc diffusion assay. An antibacterial test of both metabolite extracts produced by the most bioactive isolate UniB2439-3 was carried out following the disc-diffusion method, as described by Elshafie et al. [66] and Sofo et al. [67]. A bacterial suspension of each tested bacteria was prepared in sterile distilled water adjusted at 10^6^ CFU/mL (OD ≈ 0.2 nm) using UV-Spectrophotometer (Amersham, Ultraspec 1100 pro/500 pro, UK). A total of 4 mL of bacterial suspension mixed with soft agar 0.7% (9:1; *v*/*v*) was poured over each KB plate (Ø 9 cm). Blank discs of 6 mm (OXOID, Milan, Italy) were then placed over the plates and 15 µL from each tested metabolite extract (*Exo*-ME 16 mg/mL and *Endo*-ME 20 mg/mL) were carefully applied to the discs. Tetracycline (1600 µg/mL) was used as a positive control. The experiment was performed in triplicate, and the antibacterial activity was estimated by measuring the diameter of the inhibition zone in mm ± SDs compared to the positive control. 

### 4.5. Antibacterial Activity of Volatiles Metabolites

The tested bacterial strains were initially subcultured on 14 mL KB medium in Petri dishes and incubated at 37 °C for 24 h. The most efficient isolate of *B. bassiana* (UniB2439-3) was cultured on PDA media (14 mL Petri dishes) and incubated at 22 °C for 96 h. The test was performed according to Wan et al. [68] using a double-dish chamber containing the studied strain of *B. bassiana* in one downward dish of KB (Ø 90 mm), and the tested bacterial strains were singularly inoculated on the upward dish, either by direct colony inoculation or the spread of 50 μL of an aqueous suspension (10^7^ CFU/mL). In brief, the direct inoculation of colonies was carried out using a sterile swab to homogenize the colonies over the KB surface. The concentration of the aqueous bacterial suspension was adjusted using turbidimetry. The chamber was sealed with Parafilm™ and incubated at 37 °C in darkness for 48 h. The antibacterial activity of the eventually produced volatile metabolites was evaluated by measuring the inhibition percentages of GVC and AQS of each tested bacterial strain. The experiment was carried out twice with three replicates. 

### 4.6. SPME-GC/MS of VOCs 

The fresh culture (96 h) of the selected *Beauveria* isolate was inoculated in a glass tube of 10 mL PDA nutrient media and incubated at 22 °C for 5 days in darkness to collect the volatile organic compounds (VOCs) [69]. The eventually produced VOCs were qualitatively analyzed using the Solid Phase Micro Extraction method (SPME), as discussed below.

The SPME fiber coated with 100 μm of non-grafted poly (dimethylsiloxane) phase (Supelco 57300-U, mounted on a Supelco 57330 support; Merck KGaA, Darmstadt, Germany), was conditioned for 1 h at 250 °C in a stream of helium. A blank run was performed after each analysis to confirm that no residual compounds were polluting the fiber or the column. The fiber was later introduced to the injection port of an HP6890 plus gas chromatograph equipped with a Phenomenex Zebron ZB-5 MS capillary column (30 m × 0.25 mm ID × 0.25 μm film thickness). An HP 5973 mass-selective (mass range: 15–800 mAU; scan rate: 1.9 scan/s; EM voltage: 1435) was used as a detector, whereas helium at 0.8 mL/min was used as a carrier gas. The injection port, equipped with a glass insert (internal diameter 0.75 mm), was split at 250 °C. A desorption time of 1.0 min was used. The detector was maintained at 230 °C. Oven was maintained at 80 °C for 3 min, then the temperature was increased to 250 °C (20 °C/min) for 10 min. All the analyses were performed in triplicate. The chromatograms obtained from the total ion current were integrated without any correction for coelutions, and the results were expressed as a percent of the total area of peaks [70]. All peaks were identified from their mass spectra by comparison with those present in Wiley 275 and NIST 02 libraries [28]. PDA media (not inoculated with the fungus) was used as a negative control. The analysis was carried out twice with three replicates (different injections).

### 4.7. Statistical Analysis 

The obtained results for the biological assays were statistically analyzed, applying one-way ANOVA using the Package for the Social Sciences (SPSS) version 13.0 (Prentice Hall: Chicago, IL, USA, 2004). *Tukey* B Post-Hoc multiple comparison test was used to evaluate the significance level with a probability of *p* < 0.05.

## 5. Conclusions

*B. bassiana,* apart from being a notable entomopathogenic fungi or biocontrol agent against some phytopathogens, by itself or through its bioactive metabolites. In particular, *B. bassiana* or its bioactive metabolites could also be used efficiently to control several bacteria in the agronomic field, where the use of antibiotics is forbidden, especially in organic farming. In addition, *B. bassiana* could also be a useful biocontrol agent against MDR microorganisms to different antibiotics, which are considered a dominant medical problem worldwide. The obtained results from the current research concluded that *B. bassiana* UniB2439-3 was able to produce some interesting VOCs, such as β-elemene, which has been reported previously to have a strong antimicrobial effect against several pathogenic microorganisms. The ability of *B. bassiana* to produce the above-mentioned metabolites can underline its antagonistic activity against several phytopathogens, as reported previously in the bibliographic research. Future studies remain necessary to evaluate the in vivo antimicrobial activity of each identified bioactive VOC from *B. bassiana* against serious phytopathogens, considering that the use of antibiotics is forbidden in agriculture in many countries. Therefore, the search for possible natural alternatives as efficient antimicrobial agents remains necessary.

## Figures and Tables

**Table 1:** Antibacterial activity of diffusible metabolites from *B. bassiana* UniB2439-3.

Tested Bacteria		Diameter of Inhibition Zones (mm)		
		*Exo*-ME 16 mg/mL	*Endo*-ME 20 mg/mL	Tetracycline 1600 µg/mL
G+ve	*B. cereus*	8.5 ± 1.0 ab	0.0 ± 0.0 c	20.8 ± 1.1 b
	*B. megaterium*	10.0 ± 1.9 ab	4.0 ± 1.7 b	25.9 ± 2.3 ab
	*C. michiganensis*	12.5 ± 2.2 a	0.0 ± 0.0 c	39.5 ± 2.5 a
G−ve	*X. campestris*	9.5 ± 2.5 ab	9.0 ± 1.9 a	23.5 ± 1.7 ab
	*P. aeruginosa*	0.0 ± 0.0 c	6.5 ± 2.8 ab	10.6 ± 0.7 c
	*P. fluorescens*	6.5 ± 1.5 b	4.5 ± 1.7 b	12.3 ± 0.9 c

Values followed by different letters in each column for each tested extract against all tested bacteria are significantly different at *p* < 0.05. Data are expressed as the mean of the inhibition zone diameter (mm) for three replicates ± SDs compared to controls ± SDs.

**Table 2:** Antibacterial activity of volatile metabolites from *B. bassiana* UniB2439-3.

Tested Bacteria		Bacterial Growth Inhibition (%)		
		GVC	AQS	Tetracycline 1600 µg/mL
G+ve	*B. cereus*	35.0 ± 5.8 c	60.0 ± 5.8 c	19.5 ± 1.4 b
	*B. megaterium*	77.5 ± 2.9 a	92.0 ± 3.5 a	23.8 ± 2.1 ab
	*C. michiganensis*	55.0 ± 5.8 b	77.5 ± 2.9 b	38.5 ± 2.4 a
G−ve	*X. campestris*	27.5 ± 2.9 d	77.5 ± 8.7 b	24.3 ± 1.3 ab
	*P. aeruginosa*	37.5 ± 2.9 c	87.5 ± 2.9 a	11.2 ± 0.8 c
	*P. fluorescens*	52.5 ± 2.9 b	72.5 ± 2.9 b	13.1 ± 0.7c

(GVC) grown-visible colonies of each tested bacteria; (AQS) aqueous bacterial suspension at 10^7^ CFU/mL. Values followed by different letters in each column for each tested extract against all tested bacteria are significantly different at *p* < 0.05. Data are expressed as the mean of inhibition zone diameter (mm) for three replicates ± SDs compared to controls ± SDs.

**Table 3:** SPME-GC/MS analysis of VOCs extracted from *B. bassiana* UniB2439-3.

Compound ^a^	R.T. ^b^ (min)	R.A. ^c^ (%)	M.Wt ^d^ (g/mol)	Formula	CAS No.	Probability ^e^ (%)	Identification ^f^
Carbon dioxide	1.11	2.75 ± 0.03	44.01	CO_2_	000124-38-9	80	NIST, IR
Benzaldehyde, 2-nitro-, diaminomethylidenhydrazone	1.21	1.24 ± 0.03	207.19	C_8_H_9_N_5_O_2_	102632-31-5	43	NIST, IR
Nitrous oxide	1.48	27.57 ± 0.04	44.013	N_2_O	010024-97-2	65	NIST, IR
Ethanol	1.57	4.69 ± 0.03	46.07	C_2_H_5_OH	000064-17-5	90	NIST, IR
Silanol, trimethyl-	1.68	0.66 ± 0.05	90.2	C_3_H_10_OSi	001066-40-6	74	NIST, IR
Acetone	1.77	0.72 ± 0.06	58.08	C_3_H_6_O	000067-64-1	79	NIST, IR
Formamide, N-methylthio	2.03	0.61 ± 0.06	75.14	C_2_H_5_N_S_	018952-41-5	63	NIST, IR
Butanal, 3-methyl-	2.71	1.32 ± 0.05	86.13	C_5_H_10_O	000590-86-3	81	NIST, IR
Butanal, 2-methyl-	2.83	0.44 ± 0.04	86.13	C_5_H_10_O	000096-17-3	90	NIST, IR
1-Butanol, 3-methyl-	3.88	3.72 ± 0.03	88.15	C_5_H_12_O	000123-51-3	83	NIST, IR
Arsenous acid, tris(trimethylsilyl) ester	4.98	1.68 ± 0.02	342.49	C_9_H_27_AsO_3_Si_3_	055429-29-3	70	NIST, IR
2,4-Dimethyl-1-heptene	5.37	0.63 ± 0.03	126.24	C_9_H_18_	019549-87-2	90	NIST, IR
Heptane, 2,3-dimethyl-	5.56	0.76 ± 0.01	128.25	C_9_H_20_	003074-71-3	87	NIST, IR
Octane, 4-methyl-	5.66	1.99 ± 0.03	128.25	C_9_H_20_	002216-34-4	93	NIST, IR
Octane, 2,3,6,7-tetramethyl-	7.30	0.19 ± 0.02	170.33	C_12_H_26_	052670-34-5	63	NIST, IR
Oxalic acid, 2-ethylhexyl nonyl ester	7.33	0.21 ± 0.03	328.5	C_19_H_36_O_4_	1000309-39-2	74	NIST, IR
Dodecane, 2,6,11-trimethyl-	7.70	0.49 ± 0.03	212.41	C_15_H_32_	031295-56-4	69	NIST, IR
Decane, 3,6-dimethyl-	7.76	5.47 ± 0.03	170.33	C_12_H_26_	017312-53-7	72	NIST, IR
Heptane, 2,4-dimethyl-	7.81	2.37 ± 0.02	128.25	C_9_H_20_	002213-23-2	79	NIST, IR
2-Undecene, 4-methyl-	7.97	1.02 ± 0.03	168.32	C_12_H_24_	091695-32-8	63	NIST, IR
Oxalic acid, isohexyl neopentyl ester	8.09	0.54 ± 0.04	244.33	C_13_H_24_O_4_	1000309-73-0	64	NIST, IR
Decane, 3,7-dimethyl-	8.15	2.37 ± 0.01	170.33	C_12_H_26_	017312-54-8	87	NIST, IR
Sulfurous acid, hexyl 2-pentyl ester	8.27	0.65 ± 0.04	236.37	C_11_H_24_O_3_S	1000309-15-6	69	NIST, IR
Decane, 2,3,5-trimethyl-	9.56	0.68 ± 0.05	184.36	C_13_H_28_	062238-11-3	80	NIST, IR
Dodecane, 2,6,10-trimethyl-	9.62	0.14 ± 0.03	212.41	C_15_H_32_	003891-98-3	72	NIST, IR
Hexadecane	9.66	0.18 ± 0.04	226.44	C_16_H_34_	000544-76-3	78	NIST, IR
Dodecane, 2,6,10-trimethyl-	9.72	0.30 ± 0.01	212.41	C_15_H_32_	003891-98-3	64	NIST, IR
Heptadecane	9.89	0.40 ± 0.03	240.5	C_17_H_36_	000629-78-7	72	NIST, IR
7-Chloro-2,3-dihydro-3-(4-N,N-dimethylaminobenzylidene)-5-phenyl-1H-1,4-benzodiazepin-2-one	10.30	0.37 ± 0.01	401.9	C_24_H_20_ClN_3_O	055056-35-4	46	NIST, IR
3,6-Dioxa-2,4,5,7-tetrasilaoctane,2,2,4,4,5,5,7,7-octamethyl-	10.39	0.20 ± 0.02	294.68	C_10_H_30_O_2_Si_4_	004342-25-0	65	NIST, IR
β-elemene ^g^	10.46	6.98 ± 0.03	204.35	C_15_H_24_	000515-13-9	91	NIST, IR
3-Hydroxybromoazepam, bis(trimethylsilyl)- deriv.	11.19	0.40 ± 0.04	476.5	C_20_H_26_BrN_3_O_2_Si_2_	1000079-50-7	72	NIST, IR
2-Amino-2-oxo-acetic acid,N-[3,4-dimethylphenyl]-, ethyl ester	11.42	0.22 ± 0.03	221.25	C_12_H_15_NO_3_	024451-17-0	77	NIST, IR
Diethyl Phthalate	11.84	0.65 ± 0.01	222.24	C_12_H_14_O_4_	000084-66-2	90	NIST, IR

^a^ Compounds are listed in order of their elution on MS capillary column; ^b^ retention indices using a homologous series of n-hydrocarbons; ^c^ relative area (R.A.) (values are mean of 3 replicates ± SDs); ^d^ molecular weight; ^e^ probability percentage of identification; ^f^ method of identification: NIST = comparison with library [28]; ^g^ cyclohexane, 1-ethenyl-1-methyl-2,4-bis(1-methylethenyl)-, [1S-(1.alpha.,2.beta.,4.beta.)]. For confirmation of the obtained results, this experiment of GC-MS analysis of VOCs was carried out twice with three replicates (different injections).

**Table 4:** The tested bacterial strains in the current study.

Bacteria Name	Collection Number	Gram Type
*B. cereus* Frankland and Frankland	UniB12421	G+ve
*B. megaterium* de Bary	UniB12421	
*C. michiganensis* (Smith) Davis	UniB3718	
*X. campestris* (Pammel) Dowson	UniB7718	G−ve
*P. aeruginosa* (Schröter) Migula	UniB02421	
*P. fluorescens* (Flügge) Migula	UniB05421	

All tested bacteria, with a collection number for each strain, are conserved in the collection of SAFE, University of Basilicata, Potenza, Italy.

## Data Availability

Not applicable.

## Supplementary Materials

The following supporting information can be downloaded at: https://www.mdpi.com/article/10.3390/plants12152854/s1, Figure S1: Evolutionary relationships of *Beauveria bassiana* and related species nucleotide sequences based on ITS region (567 bp); Figure S2: Evolutionary relationships of *Beauveria bassiana* and related species nucleotide sequences based on beta tubulin 2 gene (340 bp); Figure S3: Chromatogram of VOCs extracted from *B. bassiana* UniB2439-3; Figure S4: Mass spectra of ethanol; Figure S5: Mass spectra of Butanal, 2-methyl; Figure S6: Mass spectra of 2,4-Dimethyl-1-heptene; Figure S7: Mass spectra of Octane, 4-methyl; Figure S8: Mass spectra of β-elemene. Table S1: Antagonistic antibacterial activity of the five studied isolates of *Beauveria* sp.
