# Supplementary material for: Antimicrobial Activity of Diffusible and Volatile Metabolites Emitted by Beauveria bassiana: Chemical Profile of Volatile Organic Compounds (VOCs) Using SPME-GC/MS Analysis
Source: Plants (Basel). 2023 Aug 3;12(15):2854. doi: 10.3390/plants12152854 (PMC10421005; doi:10.3390/plants12152854)
Supplement: Supplementary file 1 [file plants-12-02854-s001.zip › plants-2488594-supplementary.pdf]

Supplementary Materials

Antimicrobial Activity of Diffusible and Volatile Metabolites Emitted by *Beauveria bassiana*: Chemical Profile of Volatile Organic Compounds (VOCs) Using SPME-GC/MS Analysis

Ippolito Camele [1,*], Sadeek A. Sadeek [2], Rocco Racioppi [3] and Hazem S. Elshafie [1,*]

[1] School of Agricultural, Forestry, Food and Environment al Sciences, University of Basilicata, Viale dell'Ateneo Lucano 10, 85100 Potenza, Italy
[2] Department of Chemistry, Faculty of Science, University of Zagazig, 44519 Zagazig, Egypt; s_sadeek@zu.edu.eg
[3] Department of Sciences, University of Basilicata, Viale dell'Ateneo Lucano 10, 85100 Potenza, Italy; rocco.racioppi@unibas.it
* Correspondence: ippolito.camele@unibas.it (I.C.); hazem.elshafie@unibas.it (H.S.E.); Tel.: +39-0971205544 (I.C.); +39-0971205498 (H.S.E.); Fax: +39-0971205503 (H.S.E.)

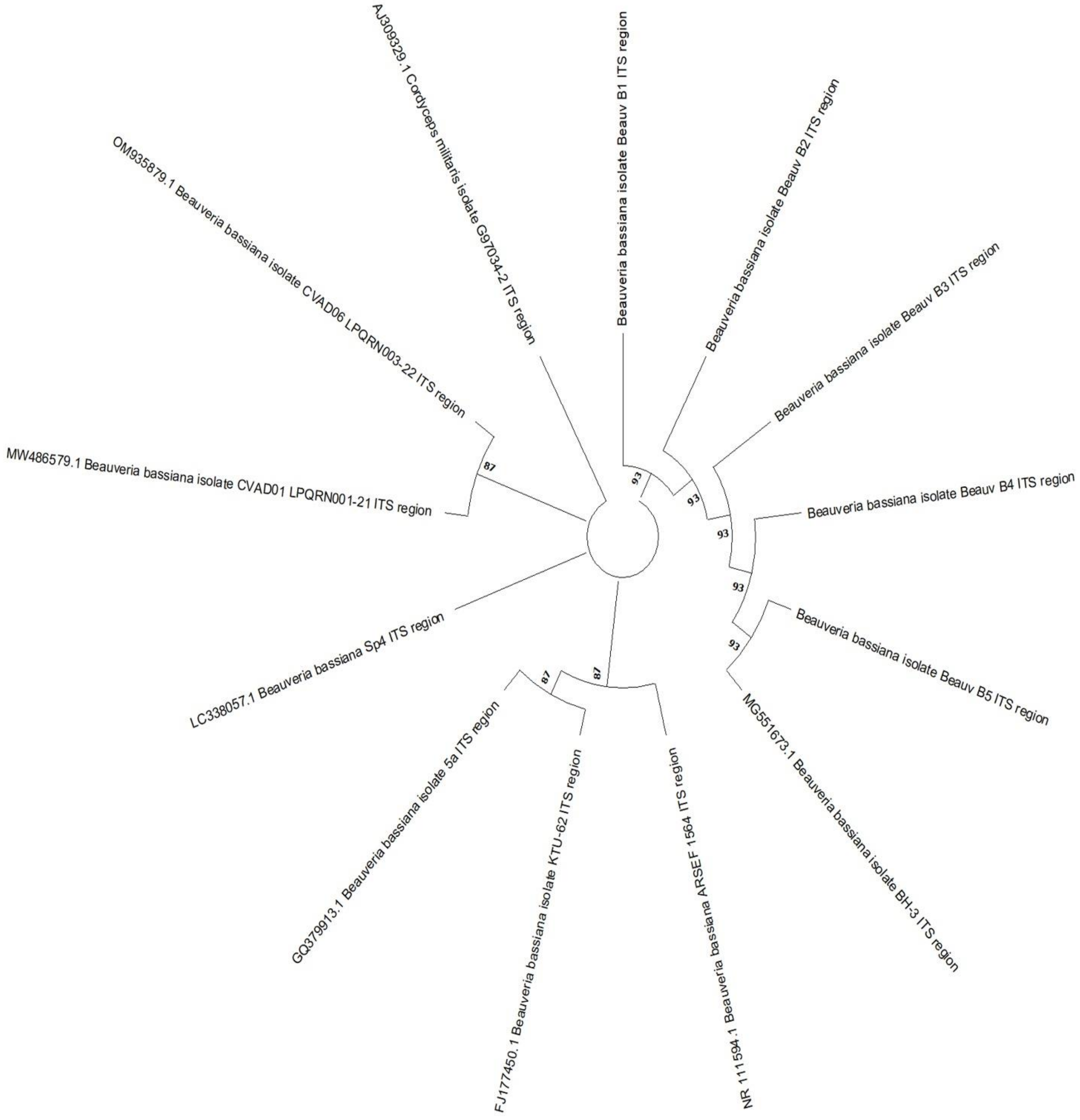

Figure S1. Evolutionary relationships of *Beauveria bassiana* and related species nucleotide sequences based on ITS region (567 bp).

The evolutionary history was inferred using the UPGMA method. The optimal tree is shown. The evolutionary distances were computed using the Maximum Composite Likelihood method and are in the units of the number of base substitutions per site. The analysis involved 13 nucleotide sequences Codon positions included were

1st+2nd+3rd+Noncoding. All ambiguous positions were removed for each sequence pair (pairwise deletion option). Evolutionary analyses were conducted in MEGA11.

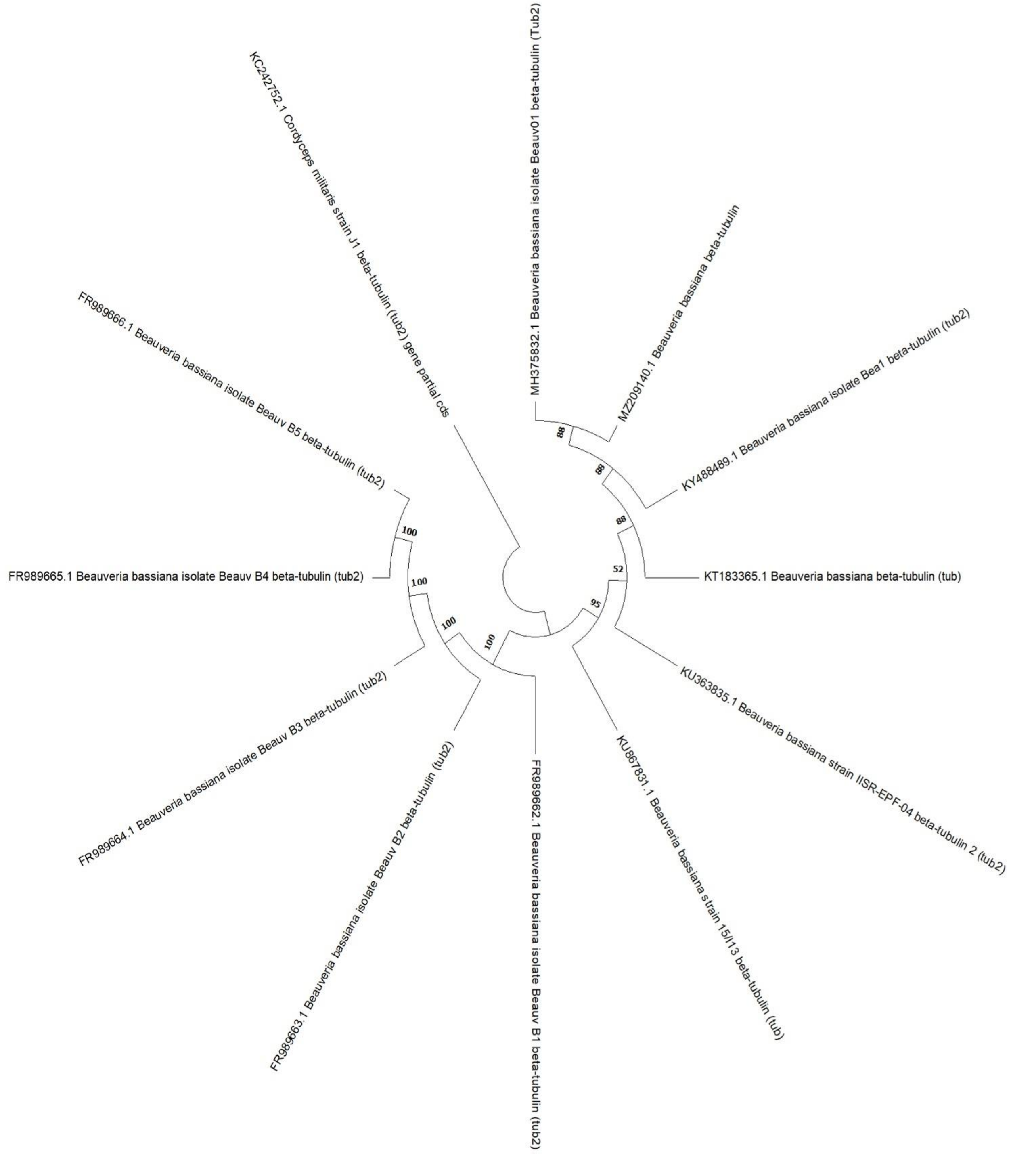

Figure S2. Evolutionary relationships of *Beauveria bassiana* and related species nucleotide sequences based on beta tubulin 2 gene (340bp).

The evolutionary history was inferred using the UPGMA method. The optimal tree is shown. The evolutionary distances were computed using the Maximum Composite Likelihood method and are in the units of the number of base substitutions per site. This analysis involved 12 nucleotide sequences. Codon positions included were

1st+2nd+3rd+Noncoding. All ambiguous positions were removed for each sequence pair (pairwise deletion option). Evolutionary analyses were conducted in MEGA11.

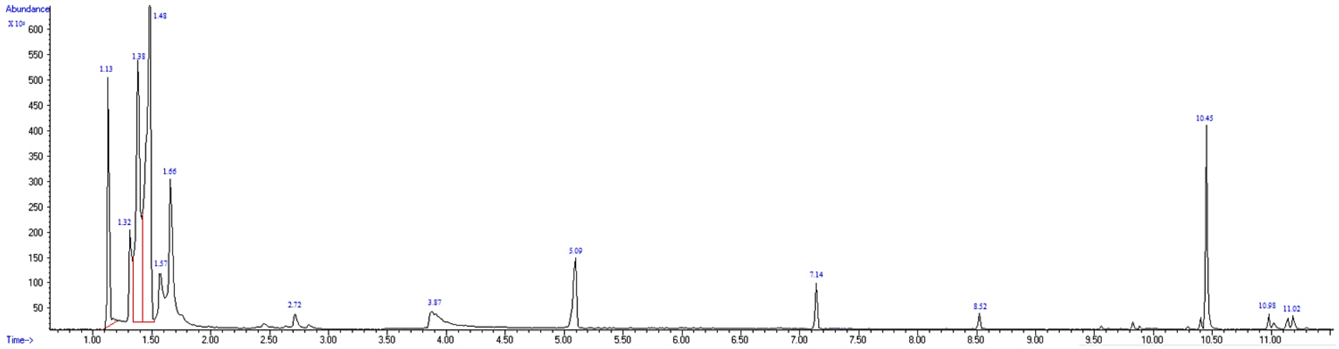

Figure S3. Chromatogram of VOCs extracted from *B. bassiana* UniB2439-3

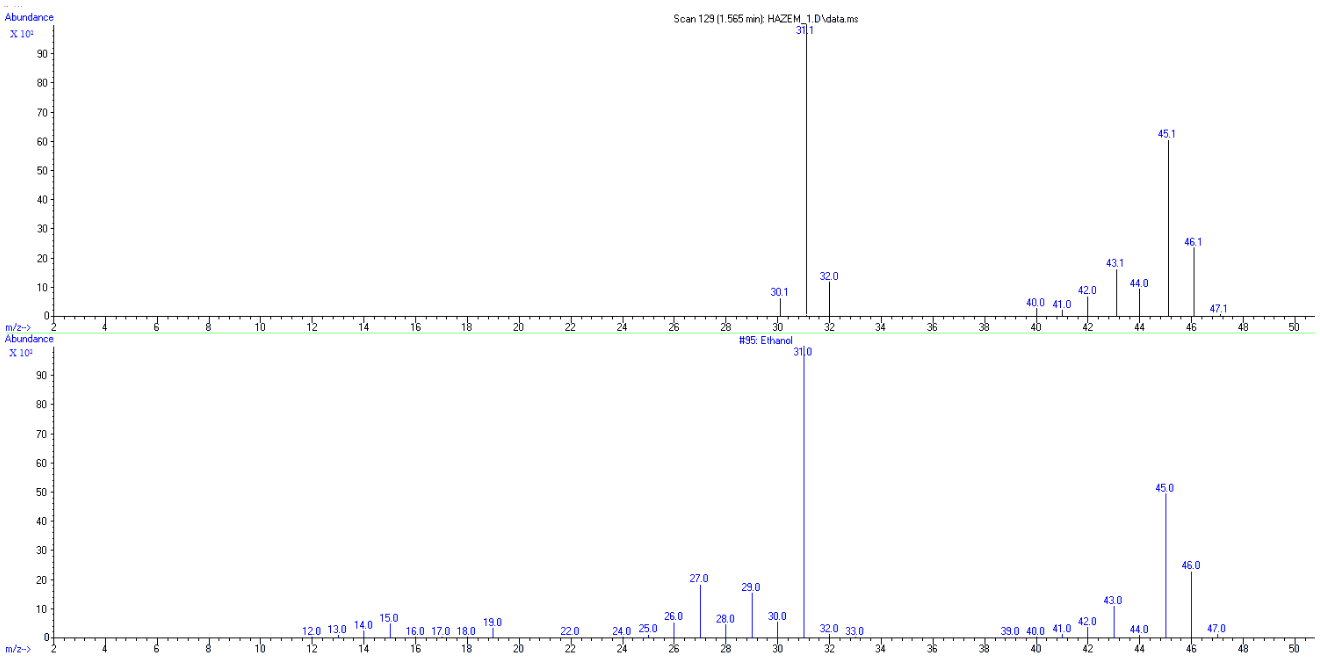

Figure S4. Mass spectra of ethanol

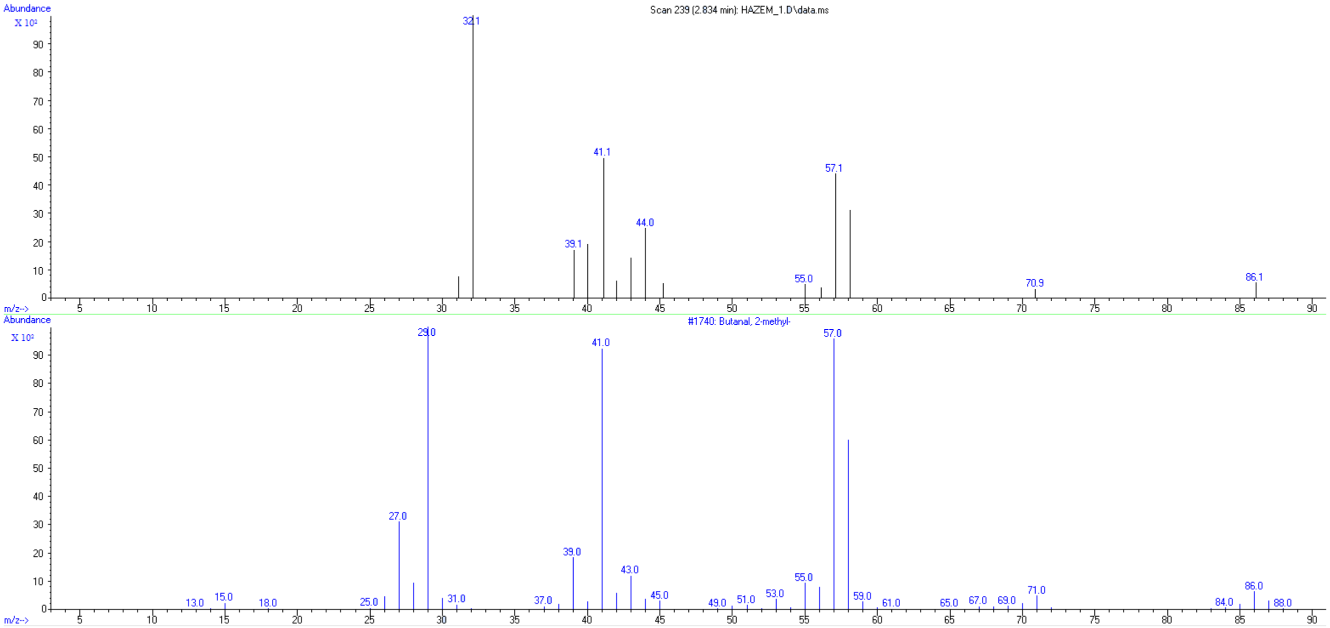

Figure S5. Mass spectra of Butanal, 2-methyl

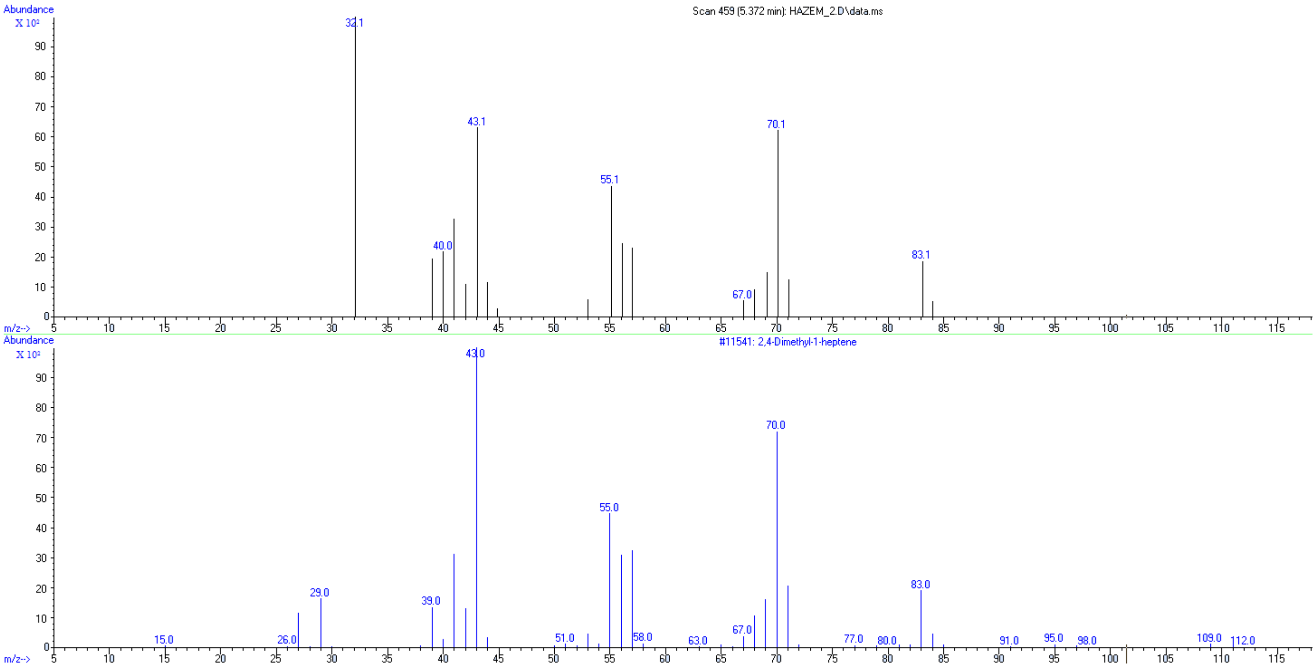

Figure S6. Mass spectra of 2,4-Dimethyl-1-heptene

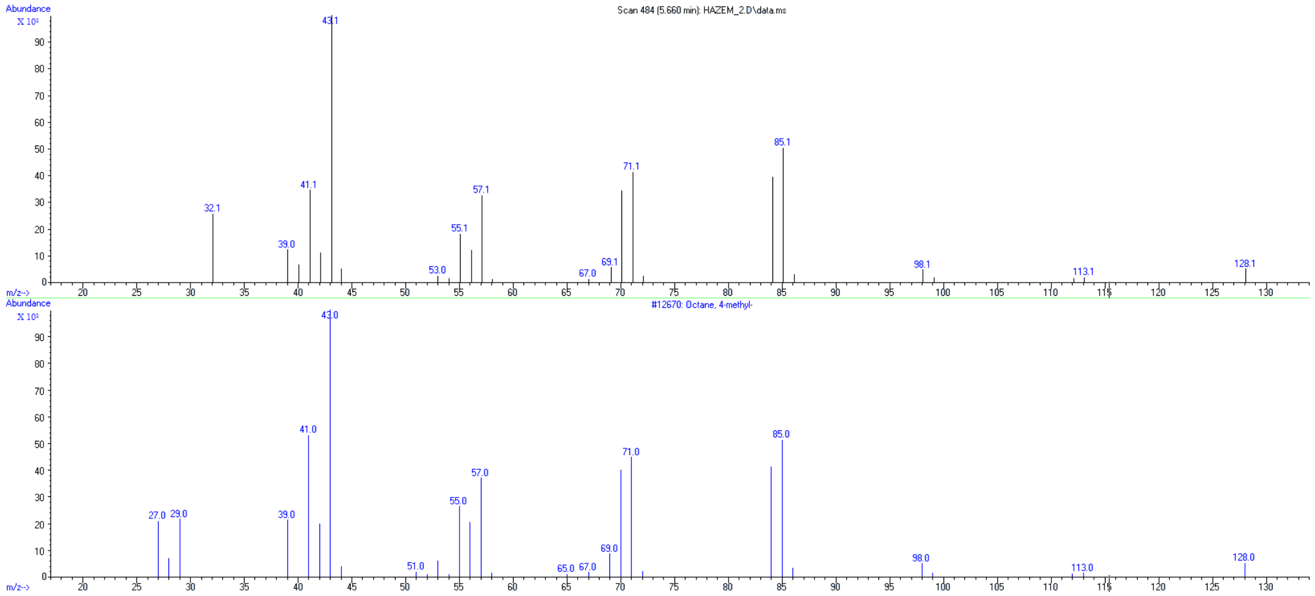

Figure S7. Mass spectra of Octane, 4-methyl

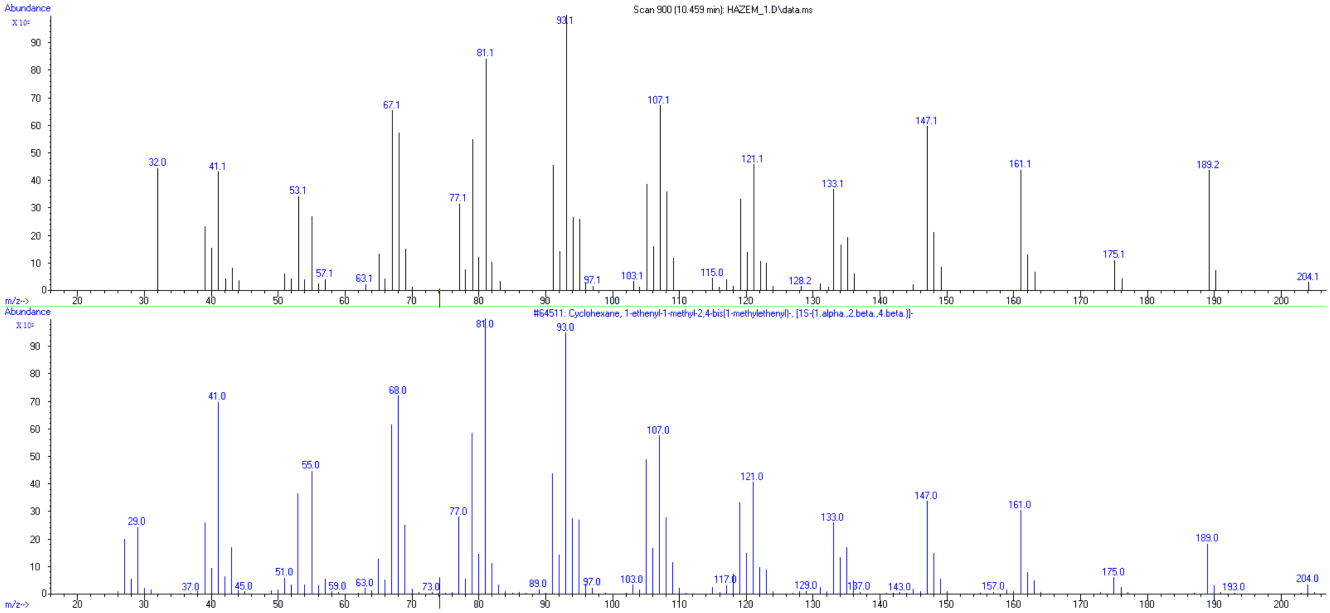

Figure S8. Mass spectra of β-elemene

Table S1. Antagonistic antibacterial activity of the five studied isolates of *Beauveria* sp.

		Diameter of inhibition zone (mm)				
		**UniB2439-1**	**UniB2439-2**	**UniB2439-3**	**UniB2439-4**	**UniB2439-5**
G+ve	*B. cereus*	10±1.2bc	12±0.8b	20±1.4a	15±1.5b	8±0.8c
	B. megaterium	8±1.4c	7±0.9c	15±3.2a	11±1.2b	10±2.4b
	B. mojavensis	12±0.2b	15±0.8b	22±2.4a	12±2.4bc	10±2.1bc
	C. michiganensis	10±3.2b	10±0.7b	19±1.4a	8±1.2c	7±1.2c
G-ve	*X. campestris*	7±1.2b	5±0.7b	10±2.2a	0±02c	5±0.7b
	X. vesicatoria	5±2.3c	10±2.2ab	12±3.2a	8±0.7ab	6±0.8b
	P. aeruginosa	0±0c	3±1.2ab	0±0c	5±1.2a	0±0c
	P. fluorescens	2±1.2b	8±0.8a	6±0.8ab	0±0c	0±0c

Values followed by different letters in each horizontal row for each tested bacteria are significantly different at $P < 0.05$ according to one-way ANOVA combined with *Tukey* B post hoc test by using SPPS program. Data are expressed as the mean of inhibition zone diameter (mm) for three
